# Prostate radiotherapy in patients with metastatic hormone-sensitive prostate cancer: A systematic review and *meta*-analysis of randomised controlled trials

**DOI:** 10.1016/j.ctro.2025.101009

**Published:** 2025-07-05

**Authors:** Navid Roessler, Marcin Miszczyk, Alessandro Dematteis, Fabio Zattoni, Tamás Fazekas, Filippo Carletti, Giuseppe Reitano, Akihiro Matsukawa, Ahmed R. Alfarhan, Angelo Cormio, Abdulrahman S. Alqahtani, Timo F.W. Soeterik, Giulia Marvaso, Giorgio Gandaglia, Péter Nyirády, Paweł Rajwa, Łukasz Nyk, Peter Soo Palencia, Michael S. Leapman, Barbara A. Jereczek-Fossa, Shahrokh F. Shariat

**Affiliations:** a Department of Urology, Comprehensive Cancer Center, Medical University of Vienna, Vienna, Austria; b Department of Urology, Medical University Center Hamburg-Eppendorf, Hamburg, Germany; c Collegium Medicum, Faculty of Medicine, WSB University, Dąbrowa Górnicza, Poland; d Department of Urology, Città della Salute e della Scienza, University of Torino School of Medicine, Torino, Italy; e Department of Surgery, Oncology, and Gastroenterology, Clinic of Urology, University of Padua, Padua, Italy; f Department of Medicine, University of Padua, Padua, Italy; g Department of Urology, Semmelweis University, Budapest, Hungary; h Centre for Translational Medicine, Semmelweis University, Budapest, Hungary; i Department of Urology, The Jikei University School of Medicine, Tokyo, Japan; j Department of Urology, Prince Saud Bin Jalawi Hospital, Al Ahsa Health Cluster, Al Ahsa, KSA; k Department of Urology, Azienda Ospedaliero-Universitaria Ospedali Riuniti Di Ancona, Università Politecnica Delle Marche, Ancona, Italy; l Department of Urology, Second Health Cluster Riyadh, Ministry of Health, KSA; m Department of Radiation Oncology, University Medical Center Utrecht, Utrecht, the Netherlands; n Department of Radiation Oncology, IEO European Institute of Oncology IRCCS, Milan, Italy; o Department of Oncology and Hemato-oncology, University of Milan, Milan, Italy; p Unit of Urology, Division of Oncology, Urological Research Institute, IRCCS San Raffaele Scientific Institute, Vita-Salute San Raffaele University, Milan, Italy; q Department of Urology, Centre of Postgraduate Medical Education, Warsaw, Poland; r Division of Surgery and Interventional Sciences, University College London, London, UK; s Robotic Surgery Center, Military Institute of Medicine, National Research Institute, Warsaw, Poland; t Department of Urology, Yale University New Haven CT USA; u Department of Urology, University of Texas Southwestern, Dallas, TX, USA; v Department of Urology, Second Faculty of Medicine, Charles University, Prague, Czech Republic; w Hourani Center for Applied Scientific Research, Al-Ahliyya Amman University, Amman, Jordan; x Karl Landsteiner Institute of Urology and Andrology, Vienna, Austria; y Research Center for Evidence Medicine, Urology Department, Tabriz University of Medical Sciences, Tabriz, Iran

**Keywords:** Radiotherapy, Local treatment, Metastatic hormone sensitive prostate cancer, Overall survival, Adverse events, Primary tumor/prostate/local treatment

## Abstract

**Introduction:**

The incidence of synchronous metastatic hormone-sensitive prostate cancer (mHSPC) is rising with the increasing use of next-generation imaging. Local radiotherapy (RT) was shown to improve survival in patients with mHSPC; however, new data require a re-assessment of the indication and value of local RT in mHSPC.

**Methods:**

In this prospectively registered systematic review and *meta*-analysis (CRD42025648251), we searched MEDLINE, Scopus, CENTRAL, and Google Scholar in March 2025 for phase 3 RCTs evaluating the addition of RT to systemic therapy to improve OS in mHSPC patients. Hazard ratios (HRs) were pooled using random-effects *meta*-analysis. Risk of Bias was assessed with Cochrane’s RoB 2 tool.

**Results:**

Out of the 10,615 individual records, we identified three RCTs: HORRAD (n = 432), STAMPEDE (n = 2,061), and PEACE-1 (n = 1,173). The systemic treatment included androgen deprivation therapy (ADT) in HORRAD, ADT ± Docetaxel in STAMPEDE, and ADT ± Docetaxel ± Abiraterone in PEACE-1 trial. Local RT was not associated with significantly improved OS in all patients (HR = 0.92; 95 % confidence interval [CI] 0.85–1.00; p = 0.06), or in those with low metastatic burden (HR = 0.74; 95 %CI 0.51–1.06; p = 0.1); however, exploratory analyses showed a significant improvement in androgen deprivation resistance-free survival (HR = 0.76; 95 %CI 0.70–0.82; p < 0.001). Local RT was associated with significant reduction in local prostate cancer related events in the HORRAD (18 % vs. 30 %) and PEACE-1 (12 % vs. 22 %) trials, but not in the STAMPEDE trial (49 % vs. 51 %).

**Conclusion:**

Local RT does not improve OS in unselected patients treated with modern systemic therapies for mHSPC. However, it delays ADT resistance and reduces local adverse events, with relatively tolerable toxicity. Future studies should refine selection criteria, ideally using PSMA-PET imaging, dynamic response markers, and/or genomic profiling, to identify mHSPC patients most likely to benefit from local RT.

## Highlights

• In the metastatic hormone-sensitive setting, local radiotherapy does not improve overall survival but significantly prolongs time to androgen deprivation resistance.
• Local radiotherapy remains a key component of multimodal therapy approaches for selected metastatic hormone-senstive prostate cancer patients.
• Redefining selection criteria is essential to better identify metastatic hormone-sensitive prostate cancer patients benefiting from local radiotherapy.

## Introduction

The incidence of synchronous metastatic hormone-sensitive prostate cancer (mHSPC) has been rising in the past decade, representing 5–8 % of newly diagnosed cases [1,2]. This can be partially attributed to the higher detection rates of new-generation imaging methods, such as PSMA-PET. At the same time, the treatment landscape of mHSPC has changed, with the combination of androgen receptor pathway inhibitor (ARPI) with androgen deprivation therapy (ADT) becoming the backbone of mHSPC management [3]. Local therapy options such as prostate radiotherapy (RT) and metastasis-directed therapy (MDT) have been gained interest, particularly in patients with low metastatic burden [[3], [4], [5]]. To date, three phase 3 randomised controlled trials (RCTs) investigated the safety and efficacy of prostate RT in mHSPC patients, but the heterogeneity in patients populations, treatment regimes, and reported results, makes their clinical interpretation challenging [[6], [7], [8], [9]]. Therefore, we aimed to synthesize the evidence on the value of local prostate RT in patients with synchronous mHSPC patients.

## Methods

This systematic review was registered with the International Prospective Register of Systematic Reviews (PROSPERO: CRD42025648251) and conducted in accordance with the Preferred Reporting Items for Systematic Reviews and Meta-analyses (PRISMA) statement (Supplementary File 1) [10]. The research question and inclusion criteria were defined using the population, intervention, comparison, outcome, and study design (PICOS) framework (Supplementary File 2). We searched MEDLINE (via PubMed), Scopus, Cochrane CENTRAL and Google Scholar for reports of RCTs that assessed the effect of adding local RT to the prostate to SOC systemic therapy on overall survival (OS). Additional endpoints included progression-free survival (PFS), androgen deprivation resistance-free survival (ADR-FS), and rates of adverse events (AEs).

The search strategy was performed in March 2025. The detailed search strategy is provided in Supplementary File 3. Records were merged and de-duplicated using the Rayyan.ai platform [11], and the title-abstract screening was conducted independently by two authors. Following title-abstract screening, full-text reports were retrieved and screened for relevance independently by two authors. Backward citation searching was performed to identify potentially relevant additional records. At each step of the review, conflicts were resolved through consensus among co-authors.

### Statistical analysis

The *meta*-analysis was performed using random-effects inverse variance method with restricted maximum-likelihood estimator, applying log-transformed HRs with corresponding standard errors as input. Heterogeneity was assessed using the *I^2^* statistic (with > 50 % considered significant) and the Cochran Q test. In cases of significant heterogeneity, differences between studies were assessed qualitatively. The outcomes were presented on forest plots as pooled HRs with corresponding 95 % confidence intervals (CI) and Z-test results. P-values < 0.05 were considered significant. All tests were two-sided.

## Risk of bias assessment

We assessed the risk of bias using the Cochrane Collaboration’s tool for assessing risk of bias [12]. Authors’ judgments about each domain for each included RCT were presented in Supplementary File 4. While all three trials were judged to have a low risk of bias overall, concerns remain regarding the measurement of the outcome: the definition varied between the trials, with OS, PFS and ADR-FS being used, which may limit comparability. Additionally, as low metastatic burden was assessed retrospectively in the HORRAD and STAMPEDE trials, the risk of bias should be considered moderate due to potential selection bias and reduced validity of subgroup analyses.

## Results

Out of 9,153 screened reports, five publications from three RCTs were included (Supplementary File 1) [[6], [7], [8]]. The HORRAD trial included 432 patients with mHSPC treated at 28 centres in the Netherlands, who were randomised to receive either ADT combined with RT to the prostate, or ADT alone [8]. The median follow-up (FU) was 47 months in the first report [8], and 75 months in a subsequent publication [13]. The STAMPEDE trial was conducted across 117 sites in UK and Switzerland, and included 2,061 patients who were randomised to receive SOC, consisting of ADT ±6 cycles Docetaxel, with or without RT to the prostate [7]. The median follow-up of the first report was 37 months [7], and 61.3 months in a latter publication [14]. Finally, the PEACE-1 trial enrolled 1,173 patients with mHSPC treated at 77 European centres, who were randomised in a 2x2 design to receive SOC therapy, consisting of ADT ±6 cycles Docetaxel ± ARPI, with or without RT to the prostate, resulting in four treatment arms [6]. The median follow-up was 72 months. The characteristics of the studies are summarised in Table 1, the used definitions are summarised in Supplementary 5.

**Table 1:** Baseline and clinical features.

Trial	HORRAD trial [8,18,30]		STAMPEDE trial [7,14]		PEACE-1 trial [6]			
Number of centers; Countries	28Netherlands		117Switzerland, UK		77Belgium, France, Ireland, Italy, Romania, Spain, Switzerland			
Number of randomized patients	432		2061		1173			
Median (IQR) follow up time (month); calculated among survivors	47 (36–68) [8]75 (63–109) [30]		37 (24–48) [7]61.3 (53.8–73.1) [14]		72 (61.2–84)			
Treatment	ADT + RT	ADT	SOC* + RT	SOC*	SOC** + RT + ARPI	SOC** + RT	SOC** + ARPI	SOC**
Number of patients	216	216	1032	1029	291	293	292	296
Age (years), median (range, IQR)	67 (62–71)	67 (61–71)	68 (63–73)	68 (63–73)	66 (IQR 60–73)		67 (IQR 60–72)	
ECOG-status*** (n, %)	0:187 (87) 1–3:29 (13)	0:176 (82) 1–3:40 (18)	0:734 (71) 1–2:298(29)	0:732 (71) 1–2:297 (29)	0:413 (70.7) 1–2:171(29.3)		0:411 (69.9) 1–2:177(30.1)	
Patients with low metastatic burden (n, %)	39(18) [16]	35(16) [16]	410 (39.7)	409 (39.7)	252 (43)		253 (43)	
Median overall survival (months)	46	39	48	N/R	N/R		N/R	
Comparison for treatment	ADT + RT vs. RT		SOC* + RT vs. SOC*		SOC** + RT +/- ARPI vs. SOC** +/- ARPI			
Overall Survival (HR, CI)	HR 0.90 95 % CI 0.70–1.14 [8] HR 0.87 95 % CI 0.69–1.11 [30]		HR 0.92 95 % CI 0.80–1.06 [7] HR 0.90 95 % CI 0.81–1.01 [14]		HR 0.98 95.1 % CI 0.83–1.14			
Overall survival in low metastatic burden (HR, CI)	HR 0.43 95 % CI 0.17–1.05 [16]		HR 0.68 95 % CI 0.52–0.91 [7] HR 0.66 95 % CI 0.54–0.82 [14]		HR 0.98 95.1 % CI 0.74–1.28			

IQR = interquartile range; ADT = androgen deprivation therapy; RT = radiotherapy; SOC = standard of care; ARPI = androgen receptor pathway inhibitors; CI = confidence interval; HR = hazard ratio; N/R = not reached; *SOC = androgen deprivation therapy +/- 6 cycles docetaxel 75 mg/m2 (after December 17, 2015), (in the RT arm, 183 (18 %) received docetaxel while in the control arm, 184 (18 %) received docetaxel); **SOC = androgen deprivation therapy +/- 6 cycles docetaxel 75 mg/m2 (in the RT +/- Abiraterone acetate 1000 mg/day + Prednisone 10 mg/day arm, 355 men (60.8 %) received docetaxel, while in the control arm +/- Abiraterone acetate 1000 mg/day + Prednisone 10 mg/day, 355 patients (60.4 %) received docetaxel); ***ECOG Performance Status Scale [31].

**Fig. 1:**
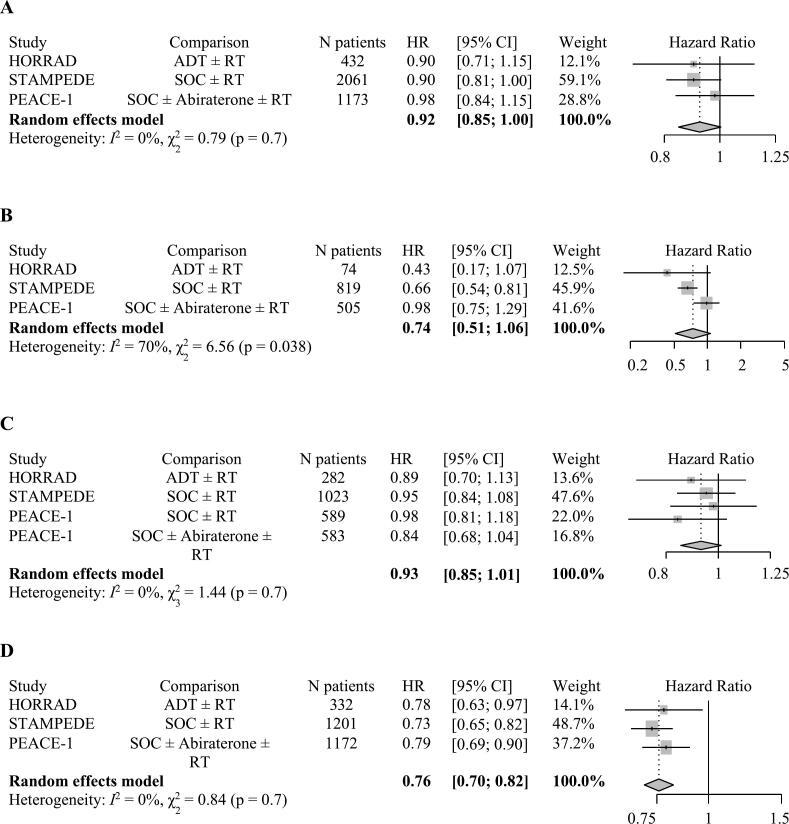
Forest-Plot − effect of local radiotherapy on A) overall survival (OS) in all included patients B) OS in patients with low metastatic burden C) progression-free survival (PFS) D) androgen deprivation resistance-free survival (ADR-FS) in metastatic hormone sensitive prostate cancer (mHSPC); ADT = androgen deprivation therapy; RT = radiotherapy; SOC = standard of care; ARPI = androgen receptor pathway inhibitors; HR = hazard ratio; CI = confidence interval.

The HR for OS in patients receiving local RT was 0.90 (95 % CI 0.70–1.14) in the primary analysis of HORRAD trial (n = 432) [8], and 0.87 (95 % CI 0.69–1.11) in the updated analysis (n = 328) [15]. In the STAMPEDE trial, the HR for OS in patients receiving local RT was 0.92 (95 % CI 0.80–1.06) in the primary analysis (n = 2,061) [7], and 0.90 (95 % CI 0.81–1.01) in the updated analysis (n = 2,061) [14]. In the PEACE-1 trial (n = 1,173), the HR for OS in patients receiving local RT was 0.98 (95.1 % CI 0.83–1.14) [6]. The pooled HR for OS of adding RT to standard of care therapy was 0.92 (95 % CI 0.85–1.00; p = 0.06) (Fig. 1 A). There was no evidence for significant heterogeneity.

In the HORRAD trial the metastatic burden was defined according to an trial-specific definition (low volume: Gleason Score < 9, fewer than five bone lesions, and prostate-specific antigen (PSA) ≤ 142 ng/mL) [16]. In the STAMPEDE and PEACE-1 trials, the CHAARTED criteria were used to define low metastatic burden (the absence of high volume: presence of visceral metastases and/or ≥ four bone metastases with at least one outside of the vertebral column and pelvis) [17]. In subgroups of patients with low metastatic burden, the HR for OS was 0.43 (95 % CI 0.17–1.05) in the HORRAD trial (n = 74) [16], 0.68 (95 % CI 0.52–0.91) in the initial (n = 819) and 0.66 (95 % CI 0.54–0.82) in the updated (n = 819) STAMPEDE trial analysis [7,14], and 0.98 (95.1 % CI 0.74–1.28) in the PEACE-1 trial (n = 505) [17]. The pooled HR for OS of adding RT to standard of care therapy in patients with low metastatic burden was 0.74 (95 % CI 0.51–1.06; p = 0.1) (Fig. 1 B). There was evidence for significant heterogeneity, which could be associated with differences in the definition of ‘low metastatic burden’ across the trials, and in the use of systemic treatments.

In the HORRAD and STAMPEDE trials, progression-free survival (PFS) was defined as the time from randomisation to first symptomatic clinical or radiological progression or death (excluding biochemical progression) [4]. The HR for PFS in the HORRAD (n = 282) and STAMPEDE (n = 1,023) trials were 0.89 (95 % CI 0.70–1.12) and 0.95 (95 % CI 0.84–1.08), respectively [4]. In the PEACE-1 trial, radiographic PFS (rPFS) was defined as the time from randomisation to the occurrence of radiographic progression or death from any cause, whichever occurred first. The HR for rPFS in the PEACE-1 trial was 0.98 (99.9 % CI 0.72–1.34) in patients receiving SOC ± RT (n = 589) and 0.84 (99.9 % CI 0.59–1.20) in patients receiving SOC + ARPI ± RT (n = 583) [6]. The pooled HR for PFS of adding RT to SOC therapy was 0.93 (95 % CI 0.85–1.01; p = 0.09) (Fig. 1 C). There was evidence for significant heterogeneity, which could be associated with differences in the definitions of the endpoints and the use of different systemic treatments.

For the ADR-FS analysis, we pooled time-to biochemical progression (BCP) from the HORRAD and STAMPEDE trials, and castration resistance-free survival (CRFS) from the PEACE-1 trial. The BCP was pooled as ADR-FS under the assumption that all patients had HSPC at baseline and were receiving ADT in the HORRAD and STAMPEDE trials. The HR for BCP was 0.78 (95 % CI 0.63–0.97) in the HORRAD trial (n = 332), and 0.73 (95 % CI 0.65–0.82) in the STAMPEDE trial (n = 1,201) [4]. The HR for CRFS in the PEACE-1 trial (n = 1,172) was 0.79 (95 % CI 0.69–0.90) for patients receiving SOC ± ARPI + RT vs. SOC ± ARPI [6]. The pooled HR for ADR-FS was 0.76 (95 % CI 0.70–0.82; p < 0.0001) (Fig. 1 D). There was evidence for significant heterogeneity, which could be associated with the different definitions of the endpoints across the studies (Supplementary File 5), and heterogenous use of systemic treatments.

In the HORRAD trial, fewer patients receiving prostate RT experienced local prostate cancer-related events (n = 30/163 (18.4 %), [ADT + RT] vs. n = 50/165 (30.3 %), [ADT]) compared to the non-RT group. The time to occurrence of local events was significantly longer in the RT group (HR 0.56, 95 % CI 0.35–0.88) [13]. In the PEACE-1 trial, the addition of prostate RT to systemic therapy decreased the number of serious genitourinary events (n = 102/458 (22.3 %), [SOC ± ARPI] vs. n = 55/451 (12.2 %), [SOC + RT ± ARPI]) [6]. In the STAMPEDE trial, 1,086 patients had at least one local intervention event (n = 530/1,086 (48.9 %), [SOC + RT] vs. n = 556 (51.1 %), [SOC]), and no significant improvement was found in patients receiving local RT (HR 0.93, 95 % CI 0.83–1.05) [14]. Additionally, the STAMPEDE trial investigated the toxicity of prostate RT using the RTOG scale. The acute toxicity during and up to 12 weeks post-radiotherapy was generally mild. Severe (grade 3 or 4) acute adverse events were rare, with 5 % (n = 43/920) of the patients experiencing severe bladder toxicity, and 1 % (n = 8/920) experiencing severe bowel toxicity. No treatment-related mortality was observed [7,14].

Both the HORRAD trial and the STAMPEDE trial investigated the effect of adding prostate RT to SOC therapy on health-related quality of life (HR-QoL) [8,14,18]. In the HORRAD trial, patients receiving RT reported significantly worse urinary symptoms (score [range 0–96] difference between the groups 11.9; 95 % CI 8.9– 14.8), bowel symptoms (score [range 0–92] difference between the groups 4.5; 95 % CI 2.1–6.8) and diarrhoea more often (score [0–100] difference between the groups 10.8; 95 % CI 7.3–14.2) than patients in the control group (all between-arm difference test p < 0.001). At 2 years, only bowel symptoms remained significantly worse (difference between the groups 8.0; 95 % CI 4.8–11.1; p < 0.001) [18]. The STAMPEDE trial showed slightly worse HR-QoL scores in the SOC + RT group at 12 weeks post randomisation (global QoL difference: −2.9 %, p = 0.003; QLQ-30 Summary Score difference: −2.0 %, p = 0.001); however, only minor differences regarding global QoL (absolute difference: −1.2 %, p = 0.050) and the QLQ-30 summary score (absolute difference: −2.0 %, p = 0.00) were observed [14]. The long-term QoL results in the PEACE-1 are not yet reported [6].

## Discussion

We observed no effect of adding prostate RT to SOC on OS in synchronous mHSPC patients, with the reduction in mortality not reaching a conventional level of statistical significance in the subset of patients with low metastatic burden. In addition, adding RT to SOC had no significant impact on PFS. However, there was a significant improvement in the ADR-FS and patients receiving RT experienced less local prostate cancer related events requiring an intervention. The treatment-related toxicity was moderate and had limited impact on health-related quality of life. These findings suggest that prostate RT should be regarded as a complementary treatment for synchronous mHSPC within a broader multimodal systemic therapy approach, where the primary consideration is balancing local PCa-related adverse events against those associated with RT. The currently used ‘low-burden’ criteria should be redefined, for defining patients likely to benefit from local therapy in synchronous mHSPC, as they do not allow to select patients who are most likely to benefit from local RT in the age of modern effective systemic therapies.

None of the included RCTs achieved their primary endpoint of OS improvement with adding local RT to SOC therapy in patients with mHSPC. Even in patients with low metastatic disease, based on the CHAARTED criteria, who are consequently recommended to consider local RT as SOC due to the findings of the STOPCAP *meta*-analysis [4], the reduction in risk of death following local RT did not reach conventional levels of statistical significance (HR 0.74; 95 % CI 0.51–1.06; p = 0.1). This difference likely reflects the influence of intensified systemic therapies, including those evaluated in the PEACE-1 trial, which may modify the relative benefit of local RT. Notably, PEACE-1 showed a PFS benefit from local RT only in low-volume patients receiving Abiraterone, indicating that the efficacy of local therapy may depend on concurrent systemic intensification [6]. Paradoxically, improved systemic control of microscopic disease could enhance the impact of local RT on long-term outcomes. While local control may have limited influence on OS in mHSPC, survival is largely driven by systemic therapies, patient factors, and tumour biology. While it is likely that many patients benefit from local RT, the currently used selection criteria need to be urgently refined. Notably, the CHAARTED criteria allow for an unlimited number of bone metastases within the axial skeleton while still classifying patients as low metastatic burden, which is clinically counterintuitive, failing to account for the actual metastatic burden or spatial distribution, which can significantly impact prognosis and treatment response. The secondary analysis of the STAMPEDE trial showed that OS decreased with the number of bone metastases increasing [19]. Such criteria could be further refined with the use of prostate-specific membrane antigen (PSMA) positron emission tomography (PET), which allows to accurately identify metastatic lesions [20], and provides additional prognostic information [21]. The proPSMA trial confirmed the enhanced diagnostic accuracy of PSMA-PET compared to standard imaging [20], and ongoing trials are assessing its potential to refine patient stratification for targeted local and metastasis-directed therapy (MDT) [22]. MDT in the setting of treatment intensification remains an investigational approach; however, it was shown to result in high rates of local control while adding minimal toxicity, warranting further investigation [5,23]. Modern predictive models could also incorporate available genetic information to help predict sensitivity to RT and identify patients with an overall favourable prognosis, who may benefit from ‘maximal’ therapy aimed at sustaining treatment response and potentially enabling de-escalation strategies [24,25]. Finally, in selected cases, local RT may be explored based on PSA and imaging response to initial therapy, with the goal of reinforcing treatment benefit in patients who respond well, allowing to further consolidate the treatment effect [21].

In all these studies, local RT was associated with better ADR-FS [4,6], resulting in a statistically and clinically significant impact in the pooled analysis (HR = 0.76; 95 % CI 0.70–0.82; p < 0.001). Considering that the definition of CRFS in the PEACE-1 trial included radiographic progression, whereas the STAMPEDE and HORRAD trials only accounted for BCP, the heterogeneity in the endpoints may potentially influence the outcomes (Supplementary File 5). Moreover, it is important to consider that the two older trials were primarily performed in the setting of ARPIs used as a later-line therapy. Currently, ADT + ARPI doublet is considered the first-line backbone SOC therapy in patients with mHSPC, with ARPI + ADT-resistance excising as an entity that necessitates salvage second-line therapies. Delaying time to ARPC is essential to ensure longevity and better QoL; local RT was not associated with PFS, which itself is associated with OS in HSPC patients [26], thereby questioning the benefit of RT. The varying definitions, the different treatment regimes, the suboptimal trial designs, and imaging methods used across the trials limit definitive conclusions. More refined selection based on newest evidence with modern imaging may help identify the optimal candidates for local therapy in the synchronous mHSPC setting.

Both the HORRAD and PEACE-1 trials demonstrated that the addition of local RT reduced local events by decreasing and delaying their occurrence [6,13], which can lead to fewer locoregional symptoms, a reduction in local interventions, and potentially fewer hospitalization-related complications, easing the burden on the healthcare system [27]. Conversely, the STAMPEDE trial did not show this beneficial effect, likely due to its broader outcome measures, including urinary tract infections and death from prostate cancer [14]. While the addition of RT may not have significantly impacted quality of life in the long term, the short-term decline observed in the initial months was manageable and improved over time [8,14,18], in agreement with previous studies [28,29]. This temporary QoL disruption must be weighed against the benefits of improved local disease control and need for interventions. The STAMPEDE trial reported relatively low rates of severe acute adverse events (5 %) [14], suggesting that the long-term benefits of RT could outweigh the short-term QoL impact. However, patient selection remains essential, as not all patients with mHSPC are likely to benefit from local RT and patient preferences should be carefully considered when deciding on treatment options in a shared decision-making process.

Several factors should be considered when interpreting the results of this systematic review and *meta*-analysis. First, the primary and secondary systemic therapies used in the included trials varied, as some of them were not yet established as SOC at the time of patient inclusion in the trial. As the PEACE1 trial showed a reduced OS benefit of RT, the value of local RT, even in low burden mHSPC, is questionable. New criteria for patient selection in the setting of modern, life-prolonging systemic therapies and modern imaging are needed. Second, there were differences in the definitions of the assessed endpoints, in particular for ADR-FS and PFS (Supplementary File 5), which introduces a bias to the pooled estimations for these. The endpoints pooled as ADR-FS for HORRAD and STAMPEDE trials did not include clinical and radiologic progression, therefore not accounting for the possibility of PSA-discordant progression. Testosterone levels were inconsistently reported, limiting the ability to assess the impact of castration status and duration on treatment outcomes. Lastly, to maximize the sample size, patient cohorts with and without modern first-line ARPI-treatment were included in the analysis, rather than focusing on a specific subgroup, as the primary goal was to evaluate the overall effect of local RT on OS in patients with mHSPC. Moreover, the impact of triplet therapy (ADT + ARPI + docetaxel) on the role of local RT remains unclear. Triplet therapy regimens were only evaluated in the STAMPEDE and PEACE-1 trials; with, only a minority of patients receiving docetaxel, limiting the generalizability of conclusions.This limits the ability to draw robust conclusions about whether local RT adds value in the setting of fully intensified systemic therapy. The effect of local RT in the setting of contemporary combination therapies remains to be determined, especially in the era of next-generation imaging, and further studies are expected to provide more clarity on this issue [4].

## Conclusions

Local RT does not significantly improve OS or PFS in unselected patients with synchronous mHSPC treated with modern systemic therapies. While a survival benefit was observed in patients with low metastatic burden, it did not reach conventional levels of statistical significance. However, local RT prolongs ADR-free survival and appears to reduce the incidence of local events requiring intervention, as reported in two of the three included studies. Given its relatively low toxicity and minimal impact on quality of life, local RT remains a potentially valuable option in selected patients. Until more robust evidence is available, local RT should be used selectively, within a shared decision-making framework that weighs its potential benefits against remaining clinicaluncertainty, and considered only in combination with modern systemic regimens, particularly ARPI-based or intensified therapies. Future studies should aim to refine patient selection, ideally by incorporating advanced diagnostic and predictivetools such as PSMA-PET imaging, dynamic treatment-response markers,and genomic or epigenetic profiling.

## Funding statement

NA (no external funding provided).

## Declaration of Competing Interest

The authors declare the following financial interests/personal relationships which may be considered as potential competing interests: Shahrokh F. Shariat received the following: Honoraria: Astellas, AstraZeneca, BMS, Ferring, Ipsen, Janssen, MSD, Olympus, Pfizer, Roche, Takeda, Consulting or Advisory Role: Astellas, AstraZeneca, BMS, Ferring, Ipsen, Janssen, MSD, Olympus, Pfizer, Pierre Fabre, Roche, Takeda, Speakers Bureau: Astellas, Astra Zeneca, Bayer, BMS, Ferring, Ipsen, Janssen, MSD, Olympus, Pfizer, Richard Wolf, Roche, Takeda, Pawel Rajwa is a paid consultant/advisor of Janssen. The other authors declare no conflicts of interest associated with this manuscript.
