# Supplementary material for: Prostate radiotherapy in patients with metastatic hormone-sensitive prostate cancer: A systematic review and meta-analysis of randomised controlled trials
Source: Clin Transl Radiat Oncol. 2025 Jul 5;54:101009. doi: 10.1016/j.ctro.2025.101009 (PMC12272608; doi:10.1016/j.ctro.2025.101009)
Supplement: Supplementary Data 1 [file mmc1.docx]

Supplementary File 1 - Preferred Reporting Items for Systematic Reviews and Meta-analyses (PRISMA) - flow diagram for new systematic reviews which included searches of databases and registers only

**Identification of studies via databases and registers**

Records identified by database searching (n = 10615):

MEDLINE: (n = 6643)

Scopus: (n = 2272)

Google Scholar (n = 1700)

Records identified by registry searching (n = 2943):

CENTRAL: (n = 2057)

Clinical trials.gov (n = 886)

Records excluded after duplicates removal

(n = 4405)

**Identification**

Records assessed for eligibility

(n = 9153)

Records excluded

(n = 9143)

Reports not retrieved

(n = 0)

Reports sought for retrieval

(n = 10)

**Screening**

Reports excluded:

Wrong study design (n = 3)

Wrong study population (n = 2)

Wrong intervention (n = 1)

Reports assessed for eligibility

(n = 10)

Report included (n = 5)

Studies included (n = 3)

**Included**

Source: Page MJ, et al. BMJ 2021;372:n71. doi: 10.1136/bmj.n71.

This work is licensed under CC BY 4.0. To view a copy of this license, visit <https://creativecommons.org/licenses/by/4.0/>

Supplementary File 2 – PICOS framework (population, intervention, comparison, outcome, and study design)

P: patients with metastatic hormone-sensitive prostate cancer (mHSPC)

I: local radiotherapy to the prostate in addition to standard-of-care systematic therapy (ADT +/- chemotherapy +/- ARPI)

C: standard of care systematic therapy alone (ADT +/- chemotherapy +/- ARPI)

O: overall survival (OS), OS in low metastatic burden, progression-free survival (PFS), androgen deprivation-resistance free-survival (ADR-FS), rates of adverse events (AEs)

S: prospective randomised controlled trials

Supplementary File 3 – Detailed Search Strategy for the databases

Pubmed: (("randomized controlled trial"[pt] OR "controlled clinical trial"[pt] OR randomized[tiab] OR placebo[tiab] OR "clinical trials as topic"[MeSH] OR randomly[tiab] OR trial[ti]) NOT (animals[mh] NOT humans[mh])) AND ("Prostatic Neoplasms"[MeSH] OR (prostat*[tiab] AND (adenocarcinoma[tiab] OR malignan*[tiab] OR cancer[tiab] OR carcinoma[tiab] OR tumo*[tiab] OR neoplas*[tiab]))) AND ("Neoplasm Metastasis"[MeSH] OR metastatic[tiab] OR "Bone Neoplasms"[MeSH] OR (osseous metastasis[tiab] OR osseous metastases[tiab]) OR ((bone[tiab] OR skelet*[tiab] OR osseous[tiab] OR osteo*[tiab]) AND metast*[tiab]) OR (metast*[tiab] AND prostat*[tiab]) OR (advanced[tiab] AND prostat*[tiab]))

Scopus: (TITLE-ABS-KEY("randomized controlled trial" OR "controlled clinical trial" OR randomized OR placebo OR "clinical trial" OR randomly OR trial)) AND (TITLE-ABS-KEY("Prostatic Neoplasms" OR "prostate cancer" OR "prostate adenocarcinoma" OR "prostate malignancy" OR "prostate tumor" OR "prostate neoplasm")) AND (TITLE-ABS-KEY("metastatic" OR "metastasis" OR "osseous metastasis" OR "bone metastasis" OR "advanced prostate cancer"))

Scholar: "clinical trial" "prostate cancer" "prostate tumor" “metastatic” "advanced prostate cancer"

Cochrane:

**1#** "Prostatic Neoplasms" OR prostat* AND (cancer OR carcinoma OR malignan* OR tumor OR tumour OR neoplas*)

**2#** metasta* OR disseminated OR "stage IV"

**3#** "Radiotherapy" OR "Proton Therapy" OR radiotherap* OR radiation OR irradia* OR chemoradi* OR "volumetric modulated arc therapy" OR tomotherapy OR proton* OR carbon-ion

**4#** #1 AND #2 AND #3

**5#** apply “trial” Filter

Clinical trials.gov: prostate cancer OR prostatic neoplasms | Other terms: metastatic OR metastasis OR advanced prostate cancer OR osseous metastasis OR bone metastasis | Phase: 3, 4 | Interventional studies

Supplementary File 4 - Table: Assessment of risk of bias [1]

| **Trial** | **Risk of bias arising from the randomisation process** | **Risk of bias due to deviations from the intended interventions (effect of assignment to intervention)** | **Missing outcome data** | **Risk of bias in measurement of the outcome** |
| --- | --- | --- | --- | --- |
| HORRAD  [2] | Randomisation was done centrally by an independent trial office Patients were assigned in a 1:1 ratio by using a restricted blockwise  **Low risk** | Randomisation done centrally by an independent trial office  Patients and investigators were aware of the study group  low metastatic burden was assessed retrospectively  **Some concerns** | All randomised patients included in analyses, not every patient was included in the follow-up due to missing data [3]  **Low risk** | Overall survival in low metastatic burden was classified by bone lesions only (via bone scintigraphy) - lead to low comparability because several lesions (visceral) could not be determined  Different definition of biochemical progression (Supplementary 5) – lead to low comparability  Different definition of progression free survival (Supplementary 5) – lead to low comparability  **Some concerns** |
| STAMPEDE  [4] | Patients were randomised centrally using a computerised algorithm  **Low risk** | Central telephone randomisation using a computer programme  Patients and investigators were aware of the study group  low metastatic burden was assessed retrospectively  **Some concerns** | All randomised patients included in analyses  **Low risk** | Not every patient received docetaxel as standard of care therapy – lead to low comparability  Different definition of biochemical progression (Supplementary 5) – lead to low comparability  Different definition of progression free survival (Supplementary 5) – lead to low comparability  **Some concerns** |
| PEACE-1  [5] | The randomisation process was performed centrally  Eligible patients were randomly assigned (1:1:1:1) using a computer algorithm  **Low risk** | The randomisation process was performed centrally using a computer algorithm  Patients and investigators were aware of the study group  **Low risk** | All randomised patients included in analyses  **Low risk** | Not every patient received docetaxel and APRI as standard of care therapy – lead to low comparability  Different definition of castration resistance-free survival (Supplementary 5) – lead to low comparability  Different definition of (radiographic) progression free survival (Supplementary 5) – lead to low comparability  **Some concerns** |

Supplementary File 5 – Definitions used in the trials

|  | HORRAD [2] | STAMPEDE [4] | PEACE-1 [5] |
| --- | --- | --- | --- |
| Overall survival (OS) | time between date of diagnosis at prostatic biopsy and date of death | time from randomisation to death from any cause | time from randomisation to death from any cause |
| Progression-free survival (PFS) | time from randomisation to first symptomatic clinical or radiological progression or death (excluding biochemical progression) [6] | time from randomisation to first symptomatic clinical or radiological progression or death (excluding biochemical progression) [6] | (radiographic) progression free survival (rPFS):  time from randomisation to the occurrence of radiographic progression or death from any cause, whichever occurred first |
| Androgen deprivation–resistant-free survival (ADR-FS) [7] | biochemical progression:  time from randomisation to first biochemical (prostate-specific antigen [PSA]) progression [6] | biochemical progression:  time from randomisation to first biochemical (prostate-specific antigen [PSA]) progression [6] | castration resistance-free survival (CRFS):  time from randomisation to the occurrence of castration-resistant prostate cancer or death from any cause, whichever occurred first; castration-resistant prostate cancer was defined as either radiographical progression or a confirmed increase in the concentration of prostate specific antigen based on three independent measurements |
| Low metastatic burden | Gleason sum score < 9, fewer than five bone leasions, prostate specific antigen (PSA) ≤ 142; the HORRAD median [8] | the absence of high volume:  presence of visceral metastases and/or ≥ four bone metastases with at least one outside of the vertebral column and pelvis [9] | the absence of high volume:  presence of visceral metastases and/or ≥ four bone metastases with at least one outside of the vertebral column and pelvis [9] |

1. Higgins JP, Altman DG, Gøtzsche PC, Jüni P, Moher D, Oxman AD, Savovic J, Schulz KF, Weeks L, Sterne JA: **The Cochrane Collaboration's tool for assessing risk of bias in randomised trials**. *Bmj* 2011, **343**:d5928.

2. Boevé LMS, Hulshof M, Vis AN, Zwinderman AH, Twisk JWR, Witjes WPJ, Delaere KPJ, Moorselaar R, Verhagen P, van Andel G: **Effect on Survival of Androgen Deprivation Therapy Alone Compared to Androgen Deprivation Therapy Combined with Concurrent Radiation Therapy to the Prostate in Patients with Primary Bone Metastatic Prostate Cancer in a Prospective Randomised Clinical Trial: Data from the HORRAD Trial**. *Eur Urol* 2019, **75**(3):410-418.

3. Boevé LMS, Hulshof M, Verhagen P, Twisk JWR, Witjes WPJ, de Vries P, Jeroen AvMR, Vis AN, van Andel G: **Prostate Cancer-related Events in Patients with Synchronous Metastatic Hormone-sensitive Prostate Cancer Treated with Androgen Deprivation Therapy with and Without Concurrent Radiation Therapy to the Prostate; Data from the HORRAD Trial**. *Eur Urol* 2024.

4. Parker CC, James ND, Brawley CD, Clarke NW, Hoyle AP, Ali A, Ritchie AWS, Attard G, Chowdhury S, Cross W *et al*: **Radiotherapy to the primary tumour for newly diagnosed, metastatic prostate cancer (STAMPEDE): a randomised controlled phase 3 trial**. *Lancet* 2018, **392**(10162):2353-2366.

5. Bossi A, Foulon S, Maldonado X, Sargos P, MacDermott R, Kelly P, Fléchon A, Tombal B, Supiot S, Berthold D *et al*: **Efficacy and safety of prostate radiotherapy in de novo metastatic castration-sensitive prostate cancer (PEACE-1): a multicentre, open-label, randomised, phase 3 study with a 2 × 2 factorial design**. *Lancet* 2024, **404**(10467):2065-2076.

6. Burdett S, Boevé LM, Ingleby FC, Fisher DJ, Rydzewska LH, Vale CL, van Andel G, Clarke NW, Hulshof MC, James ND *et al*: **Prostate Radiotherapy for Metastatic Hormone-sensitive Prostate Cancer: A STOPCAP Systematic Review and Meta-analysis**. *Eur Urol* 2019, **76**(1):115-124.

7. Oh WK, Agarwal N, Bryce A, Barata P, Bugler C, Carlsson SV, Cornell B, Dahut W, George D, Loeb S *et al*: **What's in a Name? Why Words Matter in Advanced Prostate Cancer**. *Eur Urol* 2025, **87**(2):101-103.

8. Boevé L HM, Vis A, Zwinderman K, Twisk J, Delaere K, et al. : **PD10-10 A PROSPECTIVE, RANDOMIZED CONTROLLED TRIAL EVALUATING OVERALL SURVIVAL IN PATIENTS WITH PRIMARY BONE METASTATIC PROSTATE CANCER (MPCA) RECEIVING EITHER ANDROGEN DEPRIVATION THERAPY (ADT) OR ADT COMBINED WITH CONCURRENT RADIATION THERAPY TO THE PROSTATE, FINAL DATA FROM THE HORRAD TRIAL.** . *Journal of Urology [Internet]* 2018 Apr 1, **199(4S):e231–2**.

9. Sweeney CJ, Chen YH, Carducci M, Liu G, Jarrard DF, Eisenberger M, Wong YN, Hahn N, Kohli M, Cooney MM *et al*: **Chemohormonal Therapy in Metastatic Hormone-Sensitive Prostate Cancer**. *N Engl J Med* 2015, **373**(8):737-746.
